# Gene expression profiles in neurological tissues during West Nile virus infection: a critical meta-analysis

**DOI:** 10.1186/s12864-018-4914-4

**Published:** 2018-07-13

**Authors:** Robin Kosch, Julien Delarocque, Peter Claus, Stefanie C. Becker, Klaus Jung

**Affiliations:** 1 Institute for Animal Breeding and Genetics, University of Veterinary Medicine Hannover, Foundation, Bünteweg 17p, Hanover, 30559 Germany; 2 Institute of Neuroanatomy and Cell Biology, Hannover Medical School, Carl-Neuberg-Str. 1, Hanover, 30625 Germany; 3 Institute for Parasitology, University of Veterinary Medicine Hannover, Foundation, Bünteweg 17, Hanover, 30559 Germany; 4 Research Center for Emerging Infections and Zoonoses, University of Veterinary Medicine Hannover, Foundation, Bünteweg 17, Hanover, 30559 Germany

**Keywords:** West Nile virus, Meta-analysis, Gene expression profiling, Microarray, Neuroinfectiology

## Abstract

**Background:**

Infections with the West Nile virus (WNV) can attack neurological tissues in the host and alter gene expression levels therein. Several individual studies have analyzed these changes in the transcriptome based on measurements with DNA microarrays. Individual microarray studies produce a high-dimensional data structure with the number of studied genes exceeding the available sample size by far. Therefore, the level of scientific evidence of these studies is rather low and results can remain uncertain. Furthermore, the individual studies concentrate on different types of tissues or different time points after infection. A general statement regarding the transcriptional changes through WNV infection in neurological tissues is therefore hard to make. We screened public databases for transcriptome expression studies related to WNV infections and used different analysis pipelines to perform meta-analyses of these data with the goal of obtaining more stable results and increasing the level of evidence.

**Results:**

We generated new lists of genes differentially expressed between WNV infected neurological tissues and control samples. A comparison with these genes to findings of a meta-analysis of immunological tissues is performed to figure out tissue-specific differences. While 5.879 genes were identified exclusively in the neurological tissues, 15 genes were found exclusively in the immunological tissues, and 44 genes were commonly detected in both tissues. Most findings of the original studies could be confirmed by the meta-analysis with a higher statistical power, but some genes and GO terms related to WNV were newly detected, too. In addition, we identified gene ontology terms related to certain infection processes, which are significantly enriched among the differentially expressed genes. In the neurological tissues, 17 gene ontology terms were found significantly different, and 2 terms in the immunological tissues.

**Conclusions:**

A critical discussion of our findings shows benefits but also limitations of the meta-analytic approach. In summary, the produced gene lists, identified gene ontology terms and network reconstructions appear to be more reliable than the results from the individual studies. Our meta-analysis provides a basis for further research on the transcriptional mechanisms by WNV infections in neurological tissues.

**Electronic supplementary material:**

The online version of this article (10.1186/s12864-018-4914-4) contains supplementary material, which is available to authorized users.

## Background

### Epidemiology of West Nile fever

West Nile virus [(WNV); family Flaviviridae, genus Flavivirus] is one of the most important emerging virus infections in Europe. Repeated outbreaks in Europe and the dramatic spread of the virus in the United States in the past years [1–3] have illustrated the high potential of this virus to spread globally. Global trade and travel activities further increase the risk of WNV import to formally unaffected region for example Germany and Central Europe [4, 5]. Since its first isolation in Uganda in 1937 [6], WNV has been isolated from mosquitoes in Eurasia [1] and Australia [7, 8]. Moreover, following a single introduction in New York in 1999, WNV has spread throughout the Americas [2, 3]. In nature West Nile virus is maintained in an enzootic cycle involving ornithophilic mosquitoes and birds but can infect humans, equines and other vertebrates as illustrated by repeated cases of WNV encephalitis in horses and humans [7, 9–11]. Human infection of WNV usually results in a mild febrile illness including fever, headache and fatigue which last from 1 to a few days (West Nile fever) [12]. However, severe cases with cognitive dysfunction and flaccid paralysis have also been observed [13, 14]. The initial mechanism by which WNV spreads in the body and finally reaches the central nervous system (CNS) following a mosquito bite is not completely clear. It is thought that initial replication of the virus at the bite site takes place in skin epithelial cells and regional lymph nodes. Following primary viremia, WNV spreads to the reticuloendothelial system (RES; e.g kidney, spleen) [15]. This may lead to the onset of unspecific symptoms and a secondary viremia due to replication of the virus within the RES. In rare case of CNS involvement, the virus spreads from the secondary viremia into the CNS. The mechanism by which the virus crosses the blood brain barrier (BBB) is not entirely clear, although some evidence points to an involvement of tumor necrosis factor alpha (TNF- *α*)-mediated changes in endothelial cell permeability, which enables the virus to enter the CNS [16]. Matrix Metalloproteinase 9 (MMP9) enhances the permeability of the BBB, as well [17]. In contrary to these infection promoting genes, IFN- *λ* shows protective effects by strengthening the BBB-integrity [18]. Other studies with mice indicate that infection or passive transport through the endothelium or choroid plexus epithelial cells, infection of the olfactory neurons and spread to the olfactory bulb, transport by infected immune cells, or direct axonal retrograde transport from infected peripheral neurons might also allow WNV to cross the BBB [19–22]. However, the exact mechanisms as well as the involvement of the immune response mounted by CNS cells towards WNV are still under heavy investigation (reviewed in Winkelmann et al. 2016 in [23]). The analysis of transcriptional responses initiated in different tissues after infection with WNV greatly aids further understanding of diseases development.

### High-throughput gene expression studies on West Nile virus infection

Modern technologies for biological research such as DNA microarrays [24] or high-throughput RNA-sequencing (RNA-seq) [25] allow to simultaneously measure gene expression levels of thousands of genes in biological samples. With such methods of gene expression profiling it is possible to identify genes involved in the pathogenesis of diseases and the data can contribute to the understanding of the molecular mechanisms in cells and tissues. In the context of infectious diseases it is often of interest to compare gene expression profiles between infected and non-infected individuals, between different stages after infection or between different tissues. Gene expression studies can help to better understand the role of the transcriptome in immune response [26] or immune dysregulation [27]. In the research on WNV infections many gene expression studies have been performed to correlate expression profiles with different experimental factors such as genetic background [28], infected tissue [29], time after infection [30] or expression of specific genes in the host [31].

### Meta-analysis of high-throughput gene expression studies and public databases

Individual scientific experiments or studies that are based on small sample sizes — such as many microarray or RNA-seq studies — usually have only a small statistical power and thus a limited level of scientific evidence. As a consequence, the reproducibility of study results is difficult and methods for research synthesis become more and more important [32, 33]. As one approach of research synthesis, individual studies are often combined by meta-analysis. Employing meta-analysis is widespread in the area of clinical trials and it has also become more important in the field of molecular high-throughput data. Since high-throughput data typically exhibits thousands of features (e.g. genes, mRNAs, proteins, etc.) that are observed on a much smaller number of independent biological samples, results of data analysis are even less robust than analysis of low-dimensional data [34]. Methods for meta-analysis of molecular high-throughput data were for example proposed for DNA microarray data [35, 36] or RNA-seq experiments [37]. The design of individual studies that are aggregated in meta-analyses is usually restricted to standard two-group comparisons (e.g. diseased versus control) [35]. In some rare cases, the correlation of expression data with patient survival is also considered [38]. Most of the proposed methods for meta-analysis of gene expression studies concentrate on either the combination of *p*-values, the combination of effects sizes (the log2 fold change in the case of gene expression data), or the direct merging of the individual expression data sets [36]. In contrast to meta-analysis of clinical trials, data merging is a possible approach for meta-analysis of transcriptome expression data since many journals ask their contributing authors to submit their gene expression data to public repositories such as ArrayExpress (AE) [39] or Gene Expression Omnibus (GEO) [40].

### Meta-analysis of gene expression after West Nile virus infection

In order to identify genes that show an altered expression due to WNV infection and to obtain findings with an increased level of evidence, we performed a meta-analysis of high-throughput gene expression data currently available in public repositories. So far, no such comprehensive analysis has been published. A first approach of a meta-analysis in the context of WNV infections was conducted by Lim et al. (2017) [41], which was however only a comparison of their own transcriptome expression data with external literature findings by means of Venn diagrams. They did not merge the data or individual results by statistical methods. We are further interested in finding gene ontology (GO) terms (categorizing molecular functions, biological processes and cellular components) that are linked to the selected genes which play a role in WNV infections. Therefore, we use gene set enrichment analysis (GSEA) as a further part of our meta-analysis. From the top selected genes, we derive relevance networks that characterize the correlation between these genes. We compare the network derived in the infected samples with that from the control samples. In summary, our analysis provides a holistic approach to study the transcriptome of neurological, but also immunological tissues during WNV infection. Since meta-analysis of high-dimensional expression data is not straightforward, we implement different approaches (e.g. direct merging of the expression data from the individual studies or *p*-value combination methods). We take the overlap of the results of the different approaches as final result. With this we decided to run a conservative analysis that aims to reduce false positive finding by allowing much more false negative results.

In this article, we first describe the database screening and selection of appropriate data sets, as well as the bioinformatics methods and tools for meta-analysis. The results section details the outcome of different variants of meta-analysis and compares our findings with those of the individual studies. Finally, we critically discuss our findings as well as the benefits and limitations of our meta-analyses.

## Methods

In this section, we describe the databases searches and selection process for comparable studies and detail the bioinformatics methods and approaches for meta-analysis.

### Database search and grouping of samples

The two databases AE and GEO were searched for gene expression data using the search term 〈“west nile” OR “west nile virus” OR WNV 〉. On 10-11-2017 the GEO query with this search term returned 51 studies, while the AE query returned 36 entries of which 35 were already included in the GEO results. The identified datasets reflect a variety of experimental designs and different study questions. In order to combine comparable studies, we grouped the datasets based on several criteria, including the species from which the samples were taken, the organ system the samples belong to, the type of the experiment (gene expression profiling or other high-throughput screenings) and the sample material (e.g. full organ, isolated cells or cell lines).

**Fig. 1:**
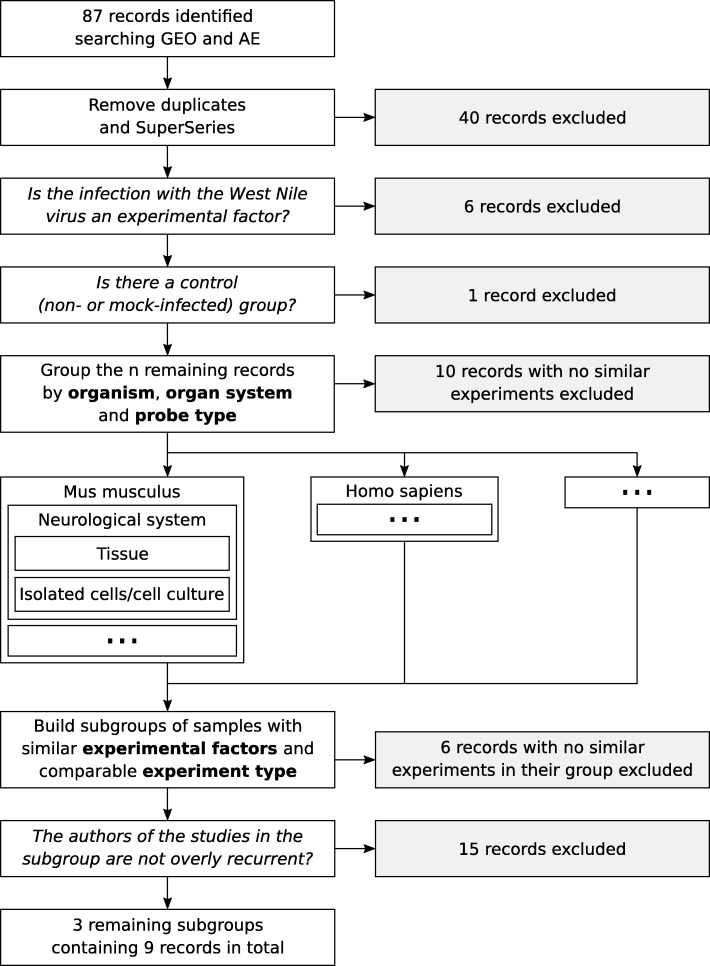
Flow diagram representing the dataset selection process. 87 records were found in total in GEO and AE using the search term 〈“west nile” OR “west nile virus” OR WNV 〉. Here a group refers to a set of records originating from the same organism and organ system with the same probe type. A subgroup is a set of records belonging to the same group and having similar experimental factors and comparable experiment type. Subgroups are refered to as groups in the paper since groups as defined here are only relevant in the selection process. In the end, 3 subgroups containing 9 records in total were considered for a meta-analysis. Horizontal arrows indicate exclusion relying on the criterion in the box they originate from. SuperSeries are entries of the GEO database that comprise multiple subseries. As the relevant subseries were already found individually by the search terms, the SuperSeries were removed

Not all samples of an individual study were necessarily used to build a group. We did not consider studies which were provided by the same authors and representing the same experiment. In these cases it could not be clarified whether the same experiment was conducted twice and which version was the correct one. When ‘time after infection’ was an experimental factor, we tried to select samples that were taken at approximately the same time as in other experiments. In the same way, cell or organism line and breed were taken into account were possible. Fig. 1 summarizes the study selection process which resulted in three suitable groups (see Table 1). These groups were all related to samples from mice. No group could be formed for human samples because the identified studies appeared to be replications from the same group of authors. For other species, the number of studies was too small to find comparable experimental settings. Group 3 is the only one using isolated cells or cell cultures. As isolated cells would enhance certain characteristics compared to a more heterogeneous cell population in whole tissues, these studies can not be included in group 2 despite originating from similar tissue types. Focusing on neurological aspects, we decided to omit group 3 from further analysis because of this lack of comparability. In contrast, group 2 was used to compare the expression profiles of the two distinct organ systems and possibly identify genes that would be specific to neurological tissues. All selected studies were based on DNA microarray measurements, none of the studies was conducted by means of RNA-seq.

**Table 1:** Groups of studies that were built after database searches and selection processes

Name	Organism	Organ system	Probe type	Included studies
group 1	Mus musculus	Neurological tissue	Tissue	5
group 2	Mus musculus	Immunological tissue	Tissue	2
group 3	Mus musculus	Immunological tissue	Isolated cells or cell culture	2

Group 1 and group 2 are based on tissue samples and were considered for meta-analysis. Group 3 was not utilized the further analysis, but mentioned here for the sake of completeness

Throughout this manuscript we use the initials of the person who uploaded the data to AE or GEO. For three out of five studies in group 1, a publication is available. Study ‘PC’ focuses on the comparison of the expression profiles after flavivirus- (WNV and JEV) and reovirus-infections [42]. The most conspicuous pathways were distinguished. In Study ‘YV’ the recovery from WNV-infections and the ensuing neurocognitive deficits were analyzed [43]. Study ‘HH’ depicted the relevance of genes involved in immune and cell death pathways after WNV- and CHIKV-infections [41]. For study ‘H1’ and ‘H2’ no publication could be found. In group 2, only study ‘MK’ was described in a publication outlining the role of RIG-I like receptors (RLR) and type I interferons (IFN) in the restriction of WNV tissue tropism [29].

### Data processing

For group 1 and 2, the raw expression data, i.e. before background correction and normalization of all samples was imported from GEO using RStudio [44] with the GEOquery package [45]. Expression data from Affymetrix chips was processed using the RMA method [46]. This method includes background correction with signal and noise close-form transformation, quantile normalization and expression level summarization using medians. We used similar processing strategies for the other types of microarrays included in the groups. Data from Agilent arrays was background-corrected with an implementation of the RMA background correction algorithm in the R-package ‘limma’ [47] and normalized using the quantile method [48]. Illumina BeadArrays data was processed using the ‘neqc’ algorithm which is very similar to RMA, except that there is no summarization step [49]. Summarization is not needed for data from Illumina and Agilent chips since they measure each transcript using a single long probe instead of many short probes targeting the same transcript as on Affymetrix chips [46, 50]. Gene transcripts represented by more than one feature are then aggregated using the median expression value. Only those genes common to all studies in the group were kept in each dataset. Samples that were not retained in the selection process because their experimental factors were not consistent with the rest of the group’s ones were removed.

Besides using the individual data sets, a merged dataset combining all individual datasets was generated for group 1 and 2. To remove batch effects in these cross-platform integrated data, the ComBat algorithm [51] was applied. For exploring grouping of samples and homogeneity between sample groups, principal component analysis (PCA) was used.

### Meta-analysis

The meta-analysis was performed in two different ways. In the first variant, differentially expressed genes were detected in the individual studies. Then a *p*-value combination method was used to combine the individual results. Here, we used the weighted inverse normal *p*-value combination implemented in the ‘MetaMA’ R-package [35] to account for the varying samples sizes between the studies. This method uses one-sided *p*-values and therefore guarantees that no genes with contradictory fold changes in the individual studies are selected. In the second analysis variant, differentially expressed genes were selected based on the merged data set. In either variant, the linear models implemented in the R-package ‘limma’ [47] were used for differential testing. Finally, resulting *p*-values were adjusted to control a false-discovery rate (FDR) of 0.01 using the procedure of Benjamini and Hochberg [52]. We call the analysis variant based on the combination of the results from the individual studies the ‘late merging’ approach, and the variant based on the merged data set the ‘early merging’ approach.

As an additional analysis, GSEA was performed to identify gene sets that play a role in WNV infections. See Subramanian et al. (2005) [53] for a detailed description of the GSEA-algorithm, based on the Kolmogorov-Smirnov test. The gene sets were derived using Gene Ontology (GO) terms [54], assigning to each gene a molecular function (MF), a biological process (BP) or a cellular component (CC). Only those gene sets for which at least two genes were available in the datasets were selected. GSEA was performed on both the list of differentially expressed genes selected in the early merging approach (i.e., the merged data set) and on the list generated in the late merging approach (i.e., merged results from individual differential analyses (DA) by *p*-value combinations). For GSEA, another analysis variant for the meta-analysis is possible. Besides the early merging of the data and the late combination of the *p*-values, an ‘intermediate’ variant can be considered. In the intermediate merging approach, the individual data sets were first analyzed to identify the differentially expressed genes and the resulting lists are directly used for GSEA. Finally, the GSEA results itself are combined by the *p*-value combination method.

In order to study how the WNV infection changes the correlation among the top selected genes, relevance networks based on the infected and control samples were derived using the R-package ‘minet’ [55]. For the network construction, only the early merged data set was used.

## Results

In this section, we describe the results of the meta-analyses performed on the studies of group 1 and group 2. First, the meta-analysis of expression data from neurological tissues is presented. Next, we describe the results of meta-analysis of immunological tissues. For both groups, we describe first the group composition and then the findings of the meta-analysis.

### Meta-analysis of group 1: neurological tissues

#### Group composition

Table 2 lists the datasets that were used for a meta-analysis of gene expression in neurological tissues during WNV infection in mice. Each of the experiments followed a two-group design comparing samples of infected subjects with those of uninfected ones. In total, 44 samples were selected for meta-analysis. Microarray platforms from three different manufacturers (Affymetrix, Agilent and Illumina) were used in these experiments. Although we tried to identify similar studies in the selection process, some heterogeneity between the studies remained. In the case of group 1, heterogeneity was incorporated by different mice lines and different times after infection of the samples. Studies H1 and H2 were published by the same authors.

**Table 2:** Studies included in group 1 (neurological tissues)

ID	Databank ID	Mice line	Tissue	Sampling time	Platform	Selected samples	Citation
H1	GSE77192	C57Bl/6	Cerebellum	6 dai	Agilent	2 × 5 (out of 60)	n.a.
H2	GSE77193	C57Bl/6	Cortex	6 dai	Agilent	2 × 5 (out of 60)	n.a.
HH	E-MTAB-5832	C57Bl/6	Cerebellum	5 dai	Affymetrix	2 × 6 (out of 29)	Lim et al. (2017)
PC	GSE53784	SW	Brain	5 to 6 dai	Affymetrix	2 × 3 (out of 12)	Clarke et al. (2014)
YV	GSE72139	C57Bl/6	Hippocampus	25 dai	Illumina	4 + 2 (out of 12)	Vasek et al. (2016)

The second column gives the databank ID from either GEO or ArrayExpress. The last column provides the number of samples selected from each study. Not each sample of a study qualified to be included into the meta-analysis. The first four studies had balances sizes of infected and control samples, study five had 4 samples in the infection group and 2 in the control group. For the last three studies, publications are available. Abbreviations: dai = days after infection; SW = Swiss Webster

#### Findings of the meta-analysis

**Fig. 2:**
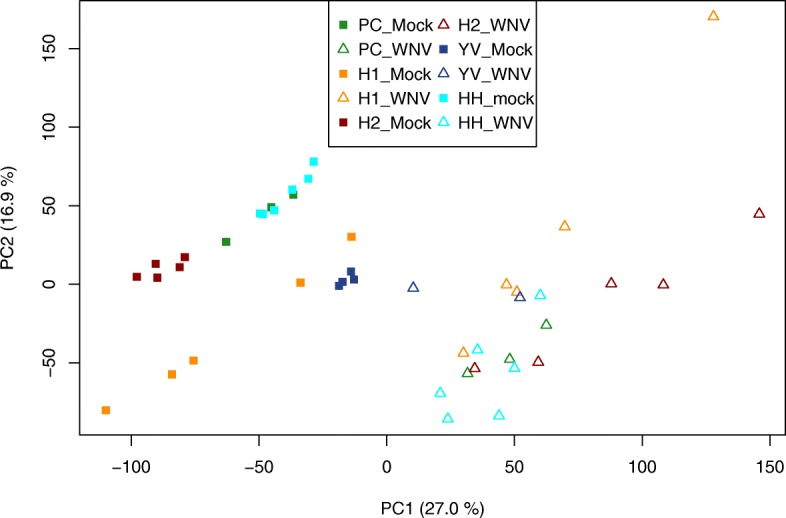
Principal component plot of the five datasets included in group 1 (neurological tissues). The axes show the first two principal components with the amount of variance these components explain from the original data. Studies are represented by different colors, WNV-infected samples are displayed as triangles, mock-infected samples as squares. The plot shows a clear separation of infected and control samples in the first principal component

**Fig. 3:**
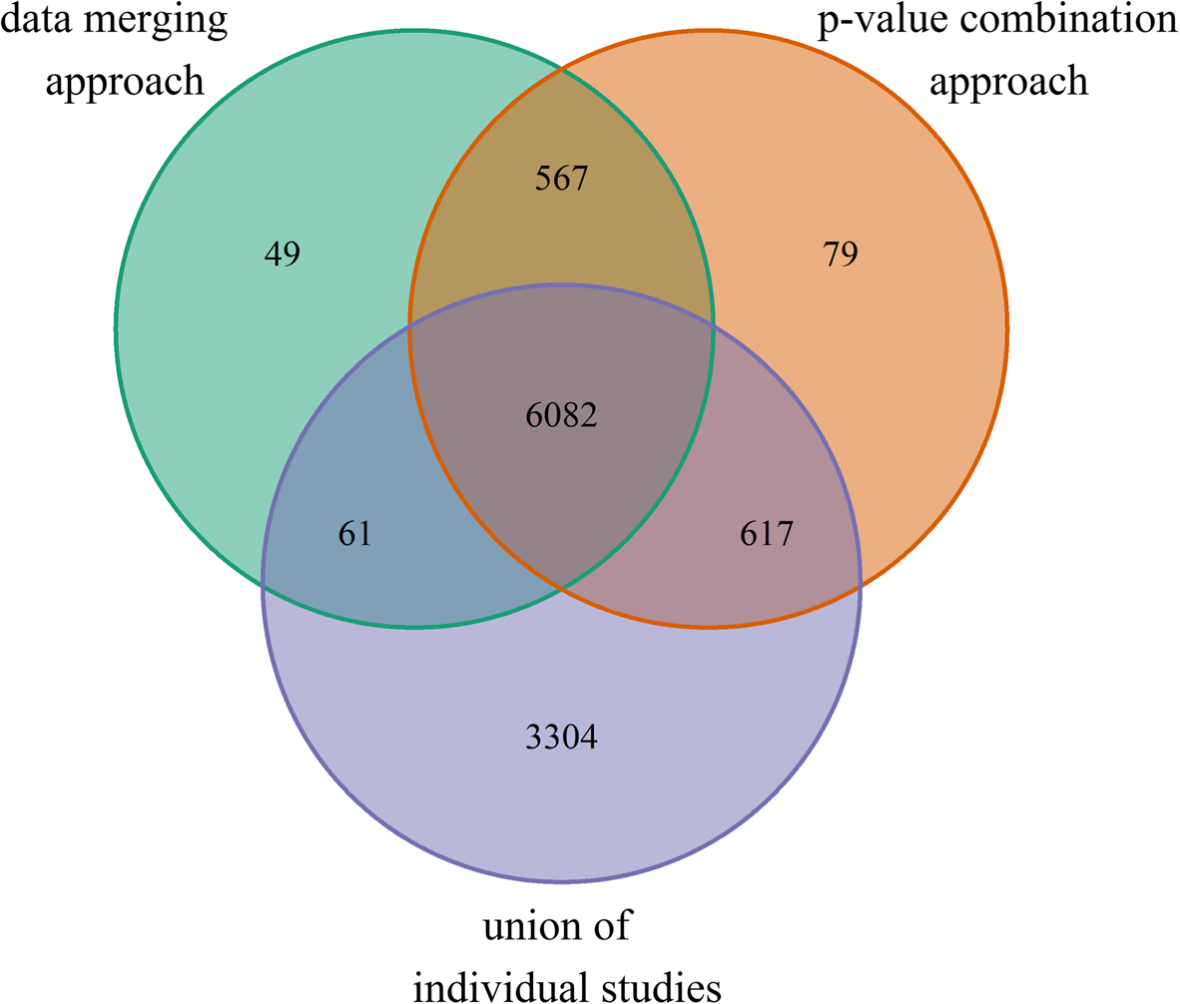
Overlap of DEGs, detected by the two approaches of meta-analysis and within the individual studies. All *p*-values have been adjusted to control a FDR of 1*%*. Approaches for meta-analysis were the direct merging of the study data (early merging) and the combination of individual results by *p*-value combination methods (late merging). Due to an increased sample size, meta-analyses had a larger power than the individual studies yielding several thousand additional findings

The analysis comprised 15.162 genes common in the 44 selected samples from all five studies. On the other hand, between 5.135 and 9.000 genes were excluded, depending on the size of the individual studies. The PCA plot shows a clear separation between the infected and the uninfected samples in the first principal component (Fig. 2). The differential analysis on the merged dataset with the ‘limma’-procedure yielded 6.759 differentially expressed (DE) genes after FDR-correction. The weighted inverse normal *p*-value combination yielded 7.345 differentially expressed genes (DEG). Figure 3 shows the overlap between the DEGs that could be detected in all of the individual studies and by either merging the data (early strategy) or by combining the *p*-values (late strategy). The great majority of DEGs, 6.082, could be identified in all three categories. 3.304 genes were found DE in the union of the individual studies, whereas 695 DEGs were only found DE in the meta-analysis variants. Slightly less DEGs (6.759) could be identified in the data merging approach compared to the 7.345 DEGs in the *p*-value combination approach.

For a more detailed analysis of specific genes, we ranked the early and late merging results by the value of their test statistic resulting from the ‘limma’ procedure, each. Test statistics have been used instead of *p*-values, because *p*-value combination can result in *p*-values of zero or bindings (*p*-values with the same value). Next, we extracted the top20 genes in each ranking (Table 3) and identified an overlap of eight genes in total. Among the two lists Rsad2 and Cd274 were the top ranked genes. We illustrate the log fold changes of Rsad2 and Cd274 in each individual study and in the merged data set by forest plots (Additional file 1: Figures A5.1 & A5.2). As can be seen in these plots, there is no disagreement regarding the direction of regulation for these genes; both genes are up-regulated in all studies. Furthermore, several of the confidence intervals overlap, indicating a good agreement between the studies. The complete lists of *p*-values and test statistics is available in Additional file 2.

**Table 3:** Top20 genes selected by the early merging strategy (first three columns) and late merging approach (last three columns) in group 1 (neurological tissues)

Gene Symbol	*t*-statistic	logFC (range)	Gene Symbol	*t*-statistic	logFC (range)
Rsad2*	41.074	5.93 − 7.72	Cd274*	14.794	5.11 − 6.34
Cxcl10*	36.524	6.30 − 8.36	Casp4	14.550	1.46 − 5.62
Cd274*	35.208	5.11 − 6.34	Ccl7	14.524	1.47 − 6.37
Ifi47	34.309	4.33 − 5.78	Samd9l*	14.360	2.83 − 4.28
Oasl2*	34.289	4.57 − 6.31	Rsad2*	14.281	5.93 − 7.72
H2-M3*	34.156	2.69 − 3.52	H2-M3*	14.113	2.69 − 3.52
Igtp	33.100	4.67 − 6.34	Cxcl10*	14.091	6.30 − 8.36
Usp18	33.080	4.46 − 6.25	Ifi207	13.920	3.54 − 4.77
Trim30a	32.723	4.18 − 5.44	Trim25*	13.915	2.40 − 3.19
Gbp3	32.571	3.76 − 5.34	Ccl3	13.882	3.54 − 6.74
Trim25*	32.195	2.40 − 3.19	Trim21*	13.863	2.88 − 3.75
Serpina3g	31.434	4.55 − 5.53	Cfb	13.846	2.32 − 6.12
Ifit3	30.856	4.17 − 6.23	Isg20	13.840	1.58 − 4.35
B2m	30.582	2.37 − 3.62	Ifi27l2a	13.821	1.58 − 6.44
Batf2	30.384	3.13 − 4.17	Irgm1	13.757	3.57 − 5.34
Phf11d	30.049	3.61 − 5.72	Rtp4	13.751	1.92 − 4.38
Tlr2	29.989	3.40 − 4.69	Irf1	13.697	2.53 − 4.13
Trim21*	29.758	2.88 − 3.75	Oasl2*	13.677	4.57 − 6.31
Samd9l*	28.802	2.83 − 4.28	Rgs1	13.610	2.77 − 5.48
Ifit2	28.146	4.28 − 5.95	AA467197	13.595	4.46 − 6.30

Gene were ranked by their *t*-statistic since the permutation *p*-values did not provide sufficient precision. For each ranked list the range of logFCs of the individual studies are given. Eight genes, flagged by *, occurred in both lists

**Fig. 4:**
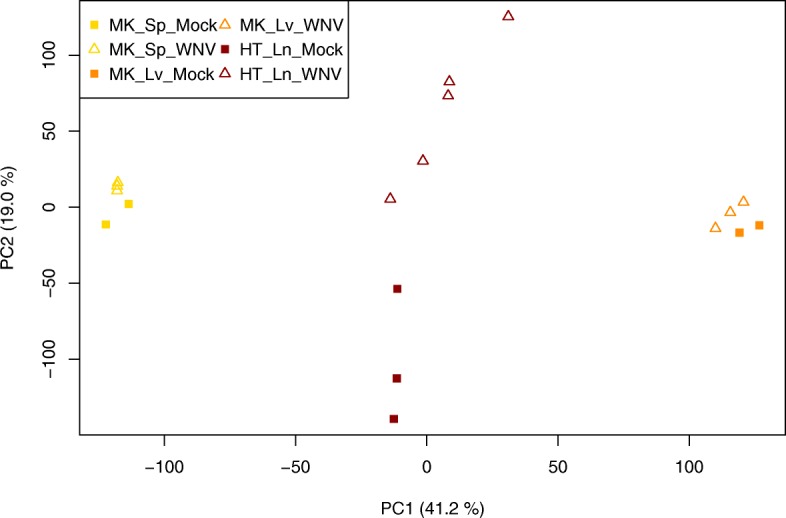
Relevance network with 8 genes selected in group 1 (neurological tissues). The network was built on the merged data set of all five studies. Red edges indicate correlations that go lost after WNF-infection, blue edges indicate new correlations after infection, and black edges indicate stable correlation in both states. Most infection-related losses can be observed for Oasl2 and the most gain are observed for H2-M3

Using the eight overlapping genes found in both top20 lists, a relevance network was calculated for the control samples and for the infected samples, based on the early merged data. In this analysis, we used only the early merging pipeline, since a late merging relevance networks from the individual studies appeared not to be reasonable. The overlap of the two resulting graphs is presented in Fig. 4. A stable correlation seems to exist between the genes Rsad2, Cxcl10 and Oasl2 (black edges). Blue edges indicate gains through the infection while red edges indicate losses. The gene with the largest number of losses is Oasl2 and the gene with the largest number of gains is H2-M3. Thus, the latter one might have a more important regulatory role in infected tissues.

Choi et al. [56] called the proportion of genes that were identified as DE in the meta-analysis but not in any of the individual studies the ‘Integration-driven Discovery Rate’ (*IDR*). In contrast the ‘Integration-driven Revision Rate’ (*IRR*) describes the percentage of genes that are declared DE in individual studies but not in meta-analysis. In the early merging analysis, these two quantities were *IDR*=9.1*%* and *IRR*=39.0*%*, respectively. For the late merging variant, the *IDR* was 8.8*%*, whereas the *IRR* was 33.4*%* (*α*=0.01).

GSEA was performed in three different variants as described above, and the intersection of significantly enriched GO terms from the three results was regarded. In total, 12.627 GO terms were studied by the three analysis pipelines. Additional file 1: Figure A5.3 shows the numbers of significantly enriched GO terms for each analysis variant, as well as their overlap. In general, the divergence between the pipelines was rather high: only 17 GO terms were found commonly in each analysis (*α*=0.01). Detailed result lists from the GSEA are provided in the Additional file 3.

### Meta-analysis of group 2: immunological tissues

#### Group composition

Table 4 lists the studies that were used for meta-analysis of gene expression in immunological tissues during West Nile virus infection in mice. Since only Agilent chips were used in the selected experiments, 20.213 genes were in common between the two studies. 987 genes were excluded from study MK; 3.949 genes from study HT. Mice lines and the sampling times were the same, too. However, the selected samples came from tissues that, despite having immunological functions, are very different from one another.

**Table 4:** Studies included in group 2 (immunological tissues)

ID	Databank ID	Mice line	Tissue	Sampling time	Platform	Selected samples	Citation
MK	GSE39259	C57Bl/6	Liver, spleen	4 dai	Agilent	2(2 + 3) (out of 40)	Suthar et al. (2013)
HT	GSE78888	C57Bl/6	Popliteal lymph node	4 dai	Agilent	3+5 (out of 51)	n.a.

The second column gives the databank ID from the GEO database. Study ‘MK’ comprises two controls and three infected samples, each for liver and spleen tissues. Abbreviation: dai = days after infection

#### Findings of the meta-analysis

**Fig. 5:**
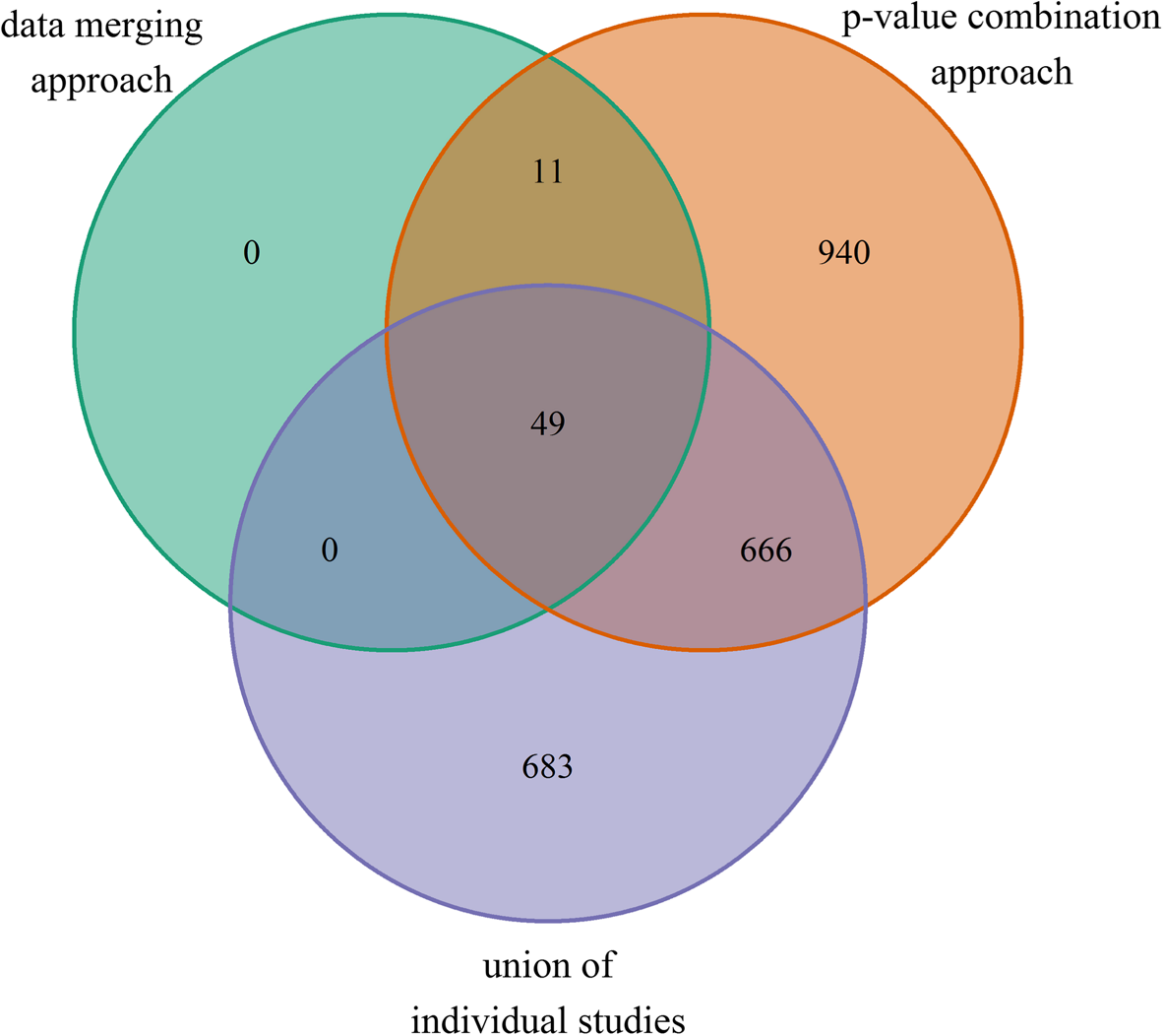
Principal component plot of the three datasets included in group 2 (immunological tissues). The axes show the first two principal components with the amount of variance these components explain from the original data. Studies are represented by different colors, WNV-infected samples are displayed as triangles, mock-infected samples as squares. Although there is a strong variance between the studies on the first principal component, there is also a separation of infected and control samples on the second principal component

**Fig. 6:**
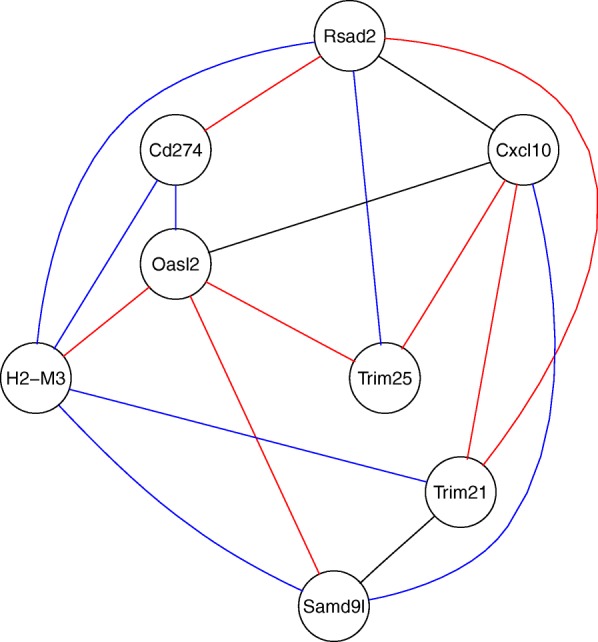
Overlap of DEGs, detected by the two approaches of meta-analysis and within the individual studies. All *p*-values have been adjusted to control a FDR of 1*%*. Approaches for meta-analysis were the direct merging of the study data (early merging) and the combination of individual results by *p*-value combination methods (late merging)

The PCA plot in Fig. 5 shows a separation between the infected and the uninfected tissues along the second principal component. Still, the samples from each organ build separate groups, which probably results from the different nature of the tissues. The differential expression analysis of the early merged dataset yielded 60 DEG after FDR-correction of the *p*-values (Fig. 6). In contrast, the weighted inverse normal *p*-value combination gives 1.666 DEGs. The overlap of both methods are 60 DEGs, as well. 683 DEGs could be detected in the union of the individual studies. Therefore, we distinguished *IDR*s of 18.3% for the early data merging approach and 57.1% for the late *p*-value combination approach. The *IRR*s were 96.5% and 48.9%. The top20 genes for the early and the late merging are listed in Table 5, five genes were commonly found in both lists. Among these five genes, Oas1a was the top-ranked gene in both lists. A forest plot for Oas1a is given in Additional file 1: Figure A5.4. Again, there is no disagreement regarding the direction of regulation. The complete lists of *p*-values and test statistics is available in Additional file 4.

**Table 5:** Top20 genes selected by the early merging strategy (first two columns) and late merging approach (last two columns) in group 2 (immunological tissues)

Gene Symbol	*t*-statistic	logFC (range)	Gene Symbol	*t*-statistic	logFC (range)
Fcgr1	12.913	1.65 − 2.28	Gzmb	8.023	2.01 − 4.29
Acod1	12.027	1.49 − 2.40	Ly6c1	7.805	1.77 − 3.58
Il10	10.055	1.15 − 1.59	Plk1	7.701	1.33 − 3.16
Oas1a*	9.377	1.95 − 2.95	Ly6a	7.693	2.09 − 2.74
Irf7*	9.340	2.27 − 3.59	Oas1a*	7.548	1.95 − 2.95
Irgm2	9.283	0.88 − 1.29	Gzma	7.547	1.98 − 4.24
Ms4a6d	9.193	1.37 − 1.48	Oas1f*	7.546	2.13 − 2.83
Oas1f*	8.670	2.13 − 2.83	Oas3*	7.514	2.50 − 2.87
Gbp6	8.635	1.06 − 2.02	Ms4a4c	7.404	0.98 − 2.48
Oas3*	8.330	2.50 − 2.87	Irf7*	7.395	2.27 − 3.59
Brip1	8.297	0.58 − 1.38	Sapcd2	7.384	1.35 − 2.15
Xaf1	8.204	1.60 − 2.27	Ifi204	7.382	1.29 − 2.10
Mlkl	8.172	0.79 − 1.38	Ly6f	7.353	1.90 − 3.11
Isg15	7.984	1.87 − 2.92	Cdk1	7.283	0.90 − 2.55
Ccl2	7.848	1.28 − 1.84	Zbp1	7.282	1.67 − 2.62
Ifit3b	7.753	1.87 − 3.38	Cdca5	7.272	1.33 − 2.89
Oasl2*	7.585	2.06 − 3.04	Oasl2*	7.266	2.06 − 3.04
Hdc	7.534	0.55 − 0.88	Ccnb2	7.252	0.98 − 2.67
Cxcl9	7.479	1.25 − 2.14	Ifitm6	7.234	0.59 − 3.06
Igtp	7.433	1.04 − 1.21	Kif22	7.219	0.76 − 2.54

Gene were ranked by their *t*-statistic since the precision of permutation *p*-values did not provide sufficient precision. For each ranked list the range of logFCs of the individual studies are given. Five genes, flagged by *, occurred in both lists

For the GSEA, we utilized 10.337 GO terms shared in both studies. The three pipelines for GSEA meta-analysis resulted in an overlap of two significantly enriched GO terms, using an *α*-error of 0.01 (Additional file 1: Figure A5.5). Detailed result lists from the GSEA are provided in the Additional file 5.

### Comparison between neurological and immunological tissues

**Fig. 7:**
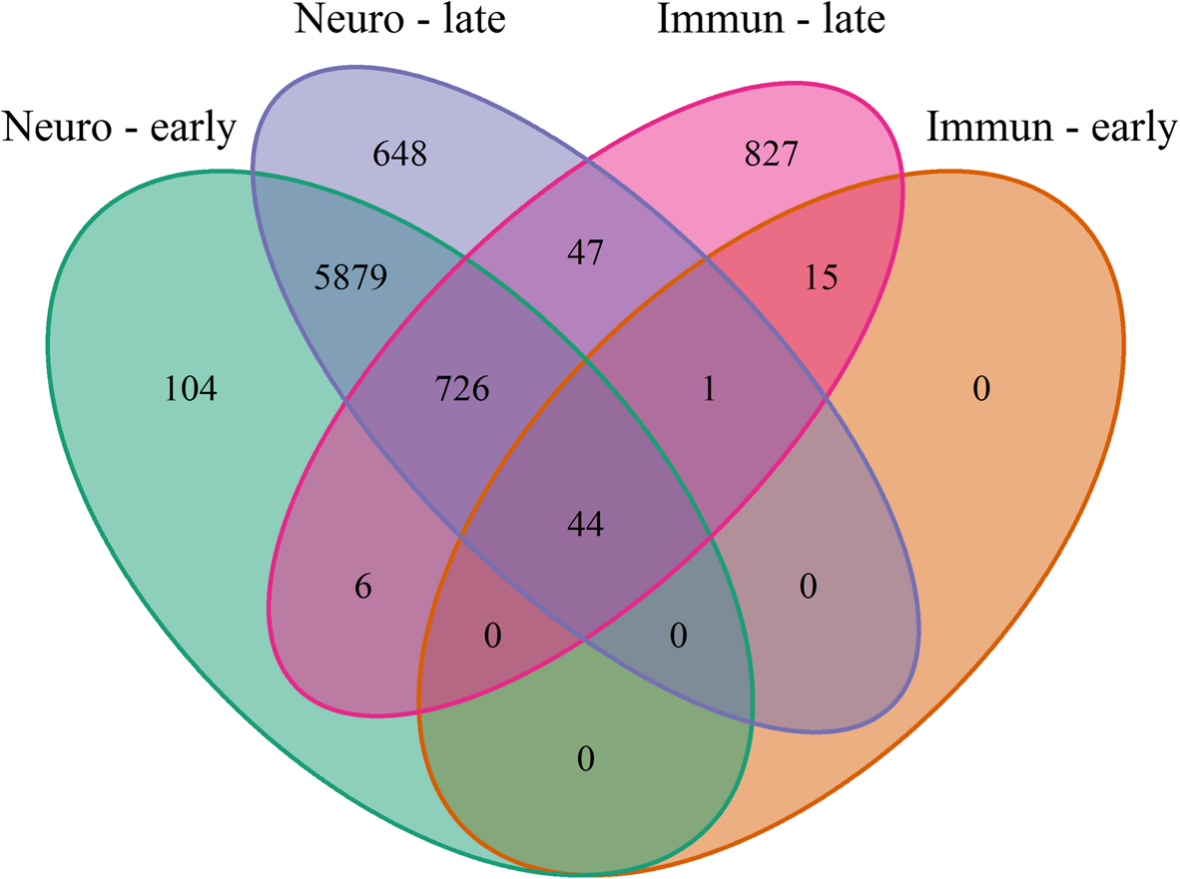
Comparison of the results of neurological and immunological tissues. Each group is further separated by early and late merging. The numbers show the amount of DEGs of each analysis variant, as well as their overlaps

Results of the differential analysis of group 1 and group 2 were compared in Fig. 7. The Venn diagram also shows the overlap of the early and late merging pipeline. Again, we regard the overlap of both pipelines as a conservative results, that gives true positive findings a higher weight by allowing for more false negatives. In the end, 44 genes could be identified as DE in the neurological and immunological tissues. On the other hand, 5.879 DEGs were found specifically in the neurological tissues; 15 DEGs were related only to the immunological tissues. Interestingly, the number of changes in the transcriptome is much larger in the neurological tissues than in the immunological ones, however this could be related to different degrees of homogeneity between the selected studies.

## Discussion

In this section, we discuss our findings in the neurological and immunological data and make a short comparison of both. Furthermore, we critically discuss the methodical issues of the meta-analysis.

### Findings in neurological tissues

In the group of neurological tissues (group 1), eight overlapping genes were found between the top20 DEGs of the early and late analysis variant were eight DEGs: Rsad2, Cxcl10, Cd274, Oasl2, H2-M3, Trim25, Trim21 and Samd9l (Table 3). The majority of these genes play a role in the innate immune system. Rsad2, Cxcl10, Samd9l and Oasl2 are involved in the type I interferon (IFN) cell signaling pathway and are therefore part of the antiviral immune system [57–59]. Rsad2 and Cxcl10 can be induced directly by IRF3 (Interferone regulatory factor) or on detours by IRF5 [57]. Rsad2, also known as Viperin, has been well described for WNV by Szretter et al. [60]. The chemokine Cxcl10 is responsible for the recruitment of CD8+ T-cells after WNV-infection [61]. CD274 (PD-L1) is a costimulatory molecule, involved in the T-cell response [62]. Trim21 and Trim25 are part of the tripartite motif family with ability to mediate ubiquitylation events. They are also induced by interferons. It has been suggested, that Oasl2 has the same effects as the Oasl in humans [58]. Oasl, as well as Trim25 are responsible for the activation, more precisely the ubiquitylation of RIG-I, which is a pattern recognition receptor, i.a. for flavivruses [63]. H2-M3 is a molecule of the MHC class Ib, known for the presentation of CD8+ T-cells. Similar biological functions could have been found for significant genes beyond the top20. We could not find a reference for the sterile alpha motif family protein 91 (Samd9l) in the context of WNV-infections. However, the SAMD9 gene is a downstream target of interferon gamma and the protein is involved in innate immunity and decreases replication of the RNA-virus Japanese Encephalitis virus [59, 64, 65]. The important role of interferons in the context of WNV infections was also described in two of the individual studies [41, 42]. The top20 genes comprise additional interferon-dependent targets, e.g. the Interferon Stimulated Exonuclease Gene 20 (Isg20). The ISG20 protein targets single-stranded RNA and displays antiviral activity towards RNA viruses [66].

The findings in the top20 DEGs match well with the results of the GSEA. Each of the 17 GO terms found in by the three analysis variants (early, intermediate or late analysis) involves a large number of genes ranging between 137 genes for the smallest GO term and 3308 for the largest. Most of these GO terms are in accordance with the results of the differential expression analysis. The GO terms GO:0048583 (regulation of response to stimulus), GO:0010033 (response to organic substance) and GO:0002684 (positive regulation of immune system process) fit to the context of WNV-infection, but are rather unspecific to allow a concrete interpretation. The same is true for the enrichment of GO term GO:0002822 (regulation of adaptive immune response based on somatic recombination of immune receptors built from immunoglobulin superfamily domains).

### Findings in immunological tissues

The findings of the meta-analysis are highly compatible with the conclusions of the study from the MK dataset [29].

While group 2 (immunological tissues) had more common DEGs among the individual datasets compared to the findings in group 1, less DEG were found both for the late merging of individual study results and in the early merging analysis strategy. This result hints at a higher heterogeneity among the datasets. However, in contrast to group 1, the *IDR* was higher than the *IRR*. Thus, one could argue that the meta-analysis had higher utility in group 2. Regarding the biological interpretation of the DA-results, a clear pattern like in group 1 could not be identified. Many of the DEGs detected in group 2 are affected by IFNs and/or have antiviral functions. Several genes from the OAS- or OASL-family (oligoadenylate synthetase), as well as genes coding for Ifit proteins (Interferon Induced proteins with Tetratricopeptide repeats) can be found [67, 68]. Again, the top20 DEGs were determined for each analysis method (early and late merging). These top20 lists overlapped in the following five genes: Oas1a, Irf7, Oas1f, Oas3 and Oasl2. Oasl2 has also been found as DEG in the neurological tissues and is described above. Except Irf7, all genes are involved in the 2’-5’-oligoadenylate synthetases. It activates the endoribonuclease RNase L, which is responsible for the degradation of viral and cellular RNAs [69]. It has been shown, that Irf7 plays a major role in the regulation of Interferone response [57].

In comparison to group 1, the results of the GSEA differ more, where the finding of the majority of detected GO terms fit to the infection context. GO:0044194 (cytolytic granule) and GO:0001730 (2’-5’-oligoadenylate synthetase activity) were significantly enriched in all three pipelines (early, intermediate, late). The importance of the 2’-5’-oligoadenylate was already depicted. Nevertheless, the overall interpretation of the GSEA-result is limited by this low number of detected gene sets in the first place.

### Comparison between neurological and immunological tissues

Regarding the top20 lists (Tables 3 and 5) Oasl2 is the only gene that is differentially expressed in the neurological tissues and in the immunological tissues. Thus, it might be that Oasl2 is a general WNV-affected gene, while the other detected DEGs are rather tissue-specific. Moreover, 43 genes were detected as DE in both tissues, beyond the top20. The much greater amount of 5.879 DEGs in neurological tissues, compared to the 15 DEGs in immunological tissues, enhances the assumption that WNV-infection leads to alterations in the brain.

### Methodical and practical issues

Meta-analysis of high-throughput expression data has the clear advantage of an increased sample size and thus an increased power to detect differentially expressed genes and enriched gene sets such as GO terms. In addition, it can be used to clarify contradictory findings regarding the direction of expression fold changes, i.e. up-regulation of a gene in one study and down-regulation in another study. Genes that are regulated in different directions in the individual studies are not selected by the early data merging approach and not by the weighted *p*-value combination approach.

Besides the benefits a meta-analysis has, it also bears some risks and has some limitations. Like in any meta-analysis it is crucial to select data sets which fit to each other regarding the study question. In the case of gene expression data, studies must focus on approximately the same type of biological samples (i.e. type of tissue), and be based on the same species. This demand may lead to a low number of final studies to be involved in the meta-analysis, even if there is a plethora of studies available before the selection process. Nevertheless, selecting fewer but more suitable studies for a meta-analysis seems to be the better choice for meaningful results.

Since the different approaches for meta-analysis (data merging or result merging) provide clearly different results, the researchers must still be careful with the final biological interpretation. Here, we have chosen a conservative approach and made only a final interpretation about the overlap of findings from different analysis pipelines. This bears, however, the risk of making false negative decisions. From our point of view, false negative conclusion can be acceptable when the goal is to obtain a stable list of top differentially expressed genes. False negatives also occur by each merging step, since genes which do not occur in all studies are omitted from analysis.

## Conclusions

Considering the benefits and limitations of meta-analyses, we think that our results provide a contribution to the knowledge about gene expression in neurological tissues after WNV infection that has a higher level of evidence than the individual studies have. In particular, the comparison with immunological tissues shows which genes may play a role after WNV infection in general, and which genes have a tissue-specific regulation.

## Additional files

Additional file 1 Additional figures A5.1-A5.5. (DOCX 420 kb)

Additional file 2 Result lists of differential analyses in group 1. The table displays result lists of the differential analyses in group 1. Rows representing those genes, common in the five individual studies of group 1. Two identifiers (‘GeneID’ and ‘Gene Symbol’) are used. Columns 3 to 18 are displaying the outcomes for different analysis variants: Columns, starting with the term ‘early’ are results from the ‘early merging’; same applies for the ‘late merging’. Columns of the individual studies begin with their identifier (see Fig. 2). For each, the *p*-values (‘.p’) and the adjusted *p*-values (‘.q’) were calculated. For the early and late merging, the absolute value of the test-statistic was also given out (‘.t.abs’). Columns ‘logFC.min’ and ‘logFC.max’ are showing the gene-wise minimum and maximum values of the logFCs from the individual studies. (XLSX 4787 kb)

Additional file 3 Result lists from GSEA analyses in group 1. The table displays result lists of the GSEA in group 1. Rows are representing those GO terms, for which at least two genes were available in each of the analysis variants. The GO-ID, as well as their specific name is given in the first column. The *p*-values (‘.p’) and adjusted *p*-values (‘.q’) are shown for each analysis variant (early, late and intermediate merging). The number of genes associated to the GO-term is presented in column ‘nPGenes’. The number of genes associated to the GO-term, which can be found in the data is presented in columns ‘early.nPGenes’ and ‘int.nPGenes’. (XLSX 1322 kb)

Additional file 4 Result lists of differential analyses in group 2. The table displays result lists of the differential analyses in group 2. Rows representing those genes, common in the two individual studies of group 2. Two identifiers (‘GeneID’ and ‘Gene Symbol’) are used. Columns 3 to 14 are displaying the outcomes for different analysis variants: Columns, starting with the term ‘early’ are results from the ‘early merging’; same applies for the ‘late merging’. Columns of the individual studies begin with their identifier (see Fig. 4). For each, the *p*-values (‘.p’) and the adjusted *p*-values (‘.q’) were calculated. For the early and late merging, the absolute value of the test-statistic was also given out (‘.t.abs’). Columns ‘logFC.min’ and ‘logFC.max’ are showing the gene-wise minimum and maximum values of the logFCs from the individual studies. (XLSX 5042 kb)

Additional file 5 Result lists from GSEA analyses in group 2. The table displays result lists of the GSEA in group 2. Rows are representing those GO terms, for which at least two genes were available in each of the analysis variants. The GO-ID, as well as their specific name is given in the first column. The *p*-values (‘.p’) and adjusted *p*-values (‘.q’) are shown for each analysis variant (early, late and intermediate merging). The number of genes associated to the GO-term is presented in column ‘nPGenes’. The number of genes associated to the GO-term, which can be found in the data is presented in columns ‘early.nPGenes’ and ‘int.nPGenes’. (XLSX 947 kb)

## Abbreviations

AE: ArrayExpress
BBB: Blood brain barrier
BP: Biological process
CC: Cellular component
CHIKV: Chikungunya virus
CNS: Central nervous system
DA: Differential analysis
DE: Differentially expressed
DEG: Differentially expressed gene
FDR: False-discovery rate
GEO: Gene expression omnibus
GSEA: Gene set enrichment analysis
GO: Gene Ontology
IDR: Integration-driven discovery rate
IFN: Interferon
IRR: Integration-driven revision rate
JEV: Japanese encephalitis virus
MF: Molecular function
MHC: Major histocompatibility complex
PCA: Principal component analysis
RES: Reticuloendothelial system
RMA: Robust multi-array average
WNV: West Nile virus
