# Supplementary material for: Gene expression profiles in neurological tissues during West Nile virus infection: a critical meta-analysis
Source: BMC Genomics. 2018 Jul 13;19:530. doi: 10.1186/s12864-018-4914-4 (PMC6044103; doi:10.1186/s12864-018-4914-4)
Supplement: Supplementary file 5 — Result lists from GSEA analyses in group 2. The table displays result lists of the GSEA in group 2. Rows are representing those GO terms, for which at least two genes were available in each of the analysis variants. The GO-ID, as well as their specific name is given in the first column. The p-values (‘.p’) and adjusted p-values (‘.q’) are shown for each analysis variant (early, late and intermediate merging). The number of genes associated to the GO-term is presented in column ‘nPGenes’. The number of genes associated to the GO-term, which can be found in the data is presented in columns ‘early.nPGenes’ and ‘int.nPGenes’. (XLSX 947 kb) [file 12864_2018_4914_MOESM5_ESM.xlsx]

**Additional file 5**

of the article

**Gene expression profiles in neurological tissues during West Nile virus infection: a critical meta-analysis**

Robin Kosch^1&^, Julien Delarocque^1&^, Peter Claus^2^, Stefanie C. Becker^3,4^, Klaus Jung^1,4*^

1) Institute for Animal Breeding and Genetics, University of Veterinary Medicine Hannover, Germany

2) Institute of Neuroanatomy and Cell Biology, Hannover Medical School, Germany

3) Institute for Parasitology, University of Veterinary Medicine Hannover, Germany

4) [Research Center for Emerging Infections and Zoonoses](http://www.tiho-hannover.de/en/clinics-institutes/institutes/research-center-for-emerging-infections-and-zoonoses/), University of Veterinary Medicine Hannover, Germany

^&^ Equal Contributor

* Corresponding author

Email address: klaus.jung@tiho-hannover.de (Klaus Jung)

**Figure A5.1 -** Forest plot of Rsad2.


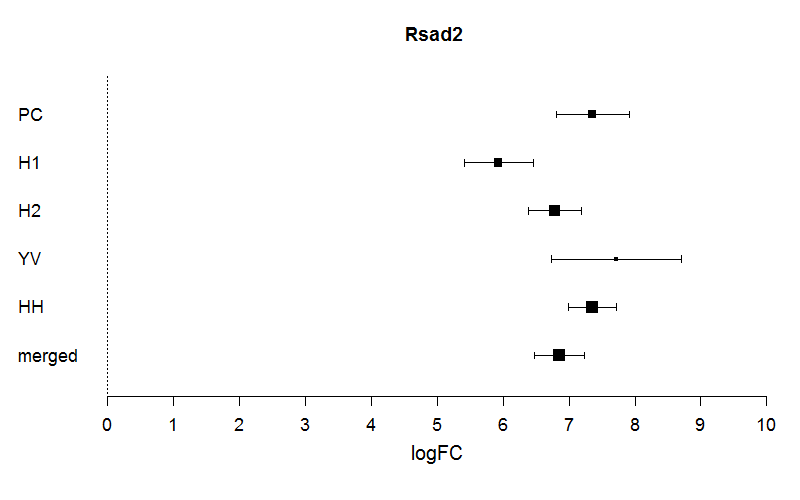


**Forest plot of Rsad2**. The plot illustrates the log fold changes of Rsad2 with 95 % confidence intervals. The log fold change derived from the merged data is printed at the bottom. The size of the squares reflects the sample size of each study. In summary, Rsad2 shows a strong up-regulation by WNV-infection in all individual studies, although there is some variance between the studies.

**Figure A5.2 -** Forest plot of Cd274.


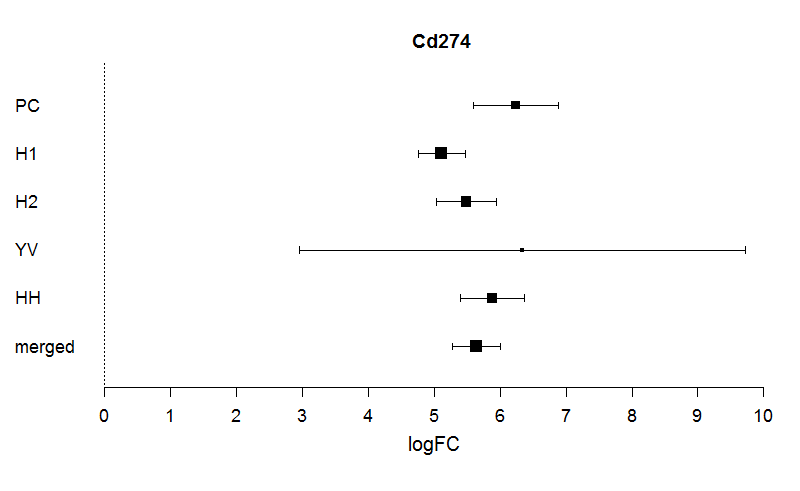


**Forest plot of Cd274.** The plot illustrates the log fold changes of Cd274 with 95 % confidence intervals. The log fold change derived from the merged data is printed at the bottom. The size of the squares reflects the sample size of each study. In summary, Cd274 shows a strong up-regulation by WNV-infection in all individual studies, although there is some variance between the studies.

**Figure A5.3 -** Overlap of selected GO terms in group 1 (neurological tissues).


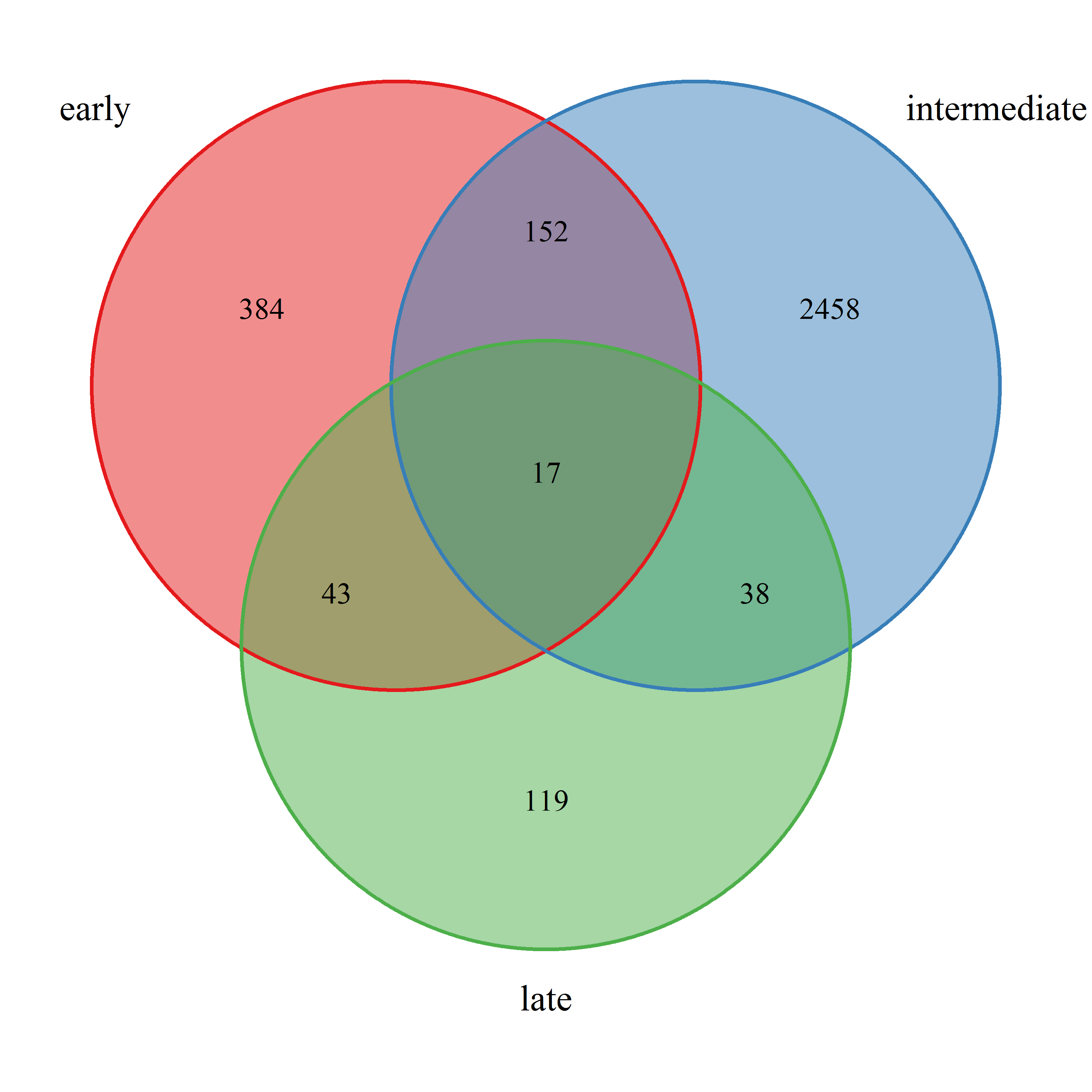


**Overlap of selected GO terms in group 1 (neurological tissues).** The three sets show the number of selected GO terms by the different variants of meta-analysis: GSEA on the merged data set (early merging), on the study-specific differential expression results (intermediate merging) and on the differential expression results after p-value combination (late merging). A p-value cutoff of 0.01 was used.

**Figure A5.4 -** Forest plot of Oas1a.


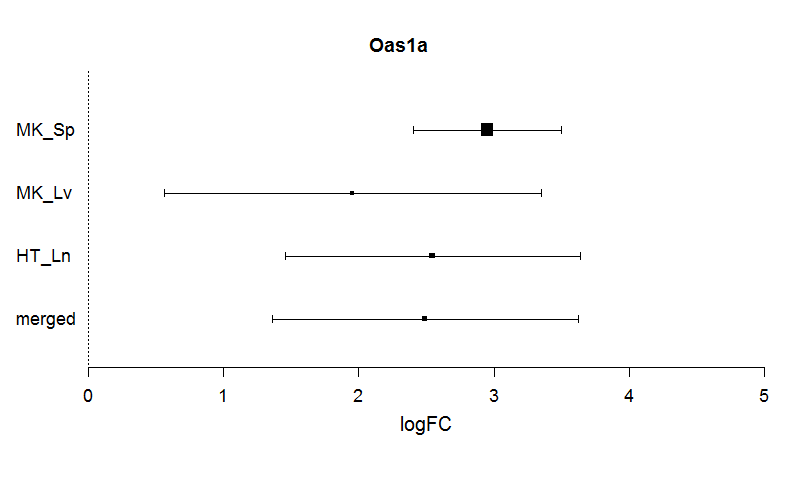


**Forest plot of Oas1a.** The plot illustrates the log fold changes of Oas1a with 95 % confidence intervals. The log fold change derived from the merged data is printed at the bottom. The size of the squares reflects the sample size of each study. In summary, Oas1a shows a strong up-regulation by WNV-infection in all individual studies, although there is some variance between the studies.

**\label{fig:Oas1a_group2_forest.png**

**Figure A5.5 -** Overlap of selected GO terms in group 2 (immunological tissues).


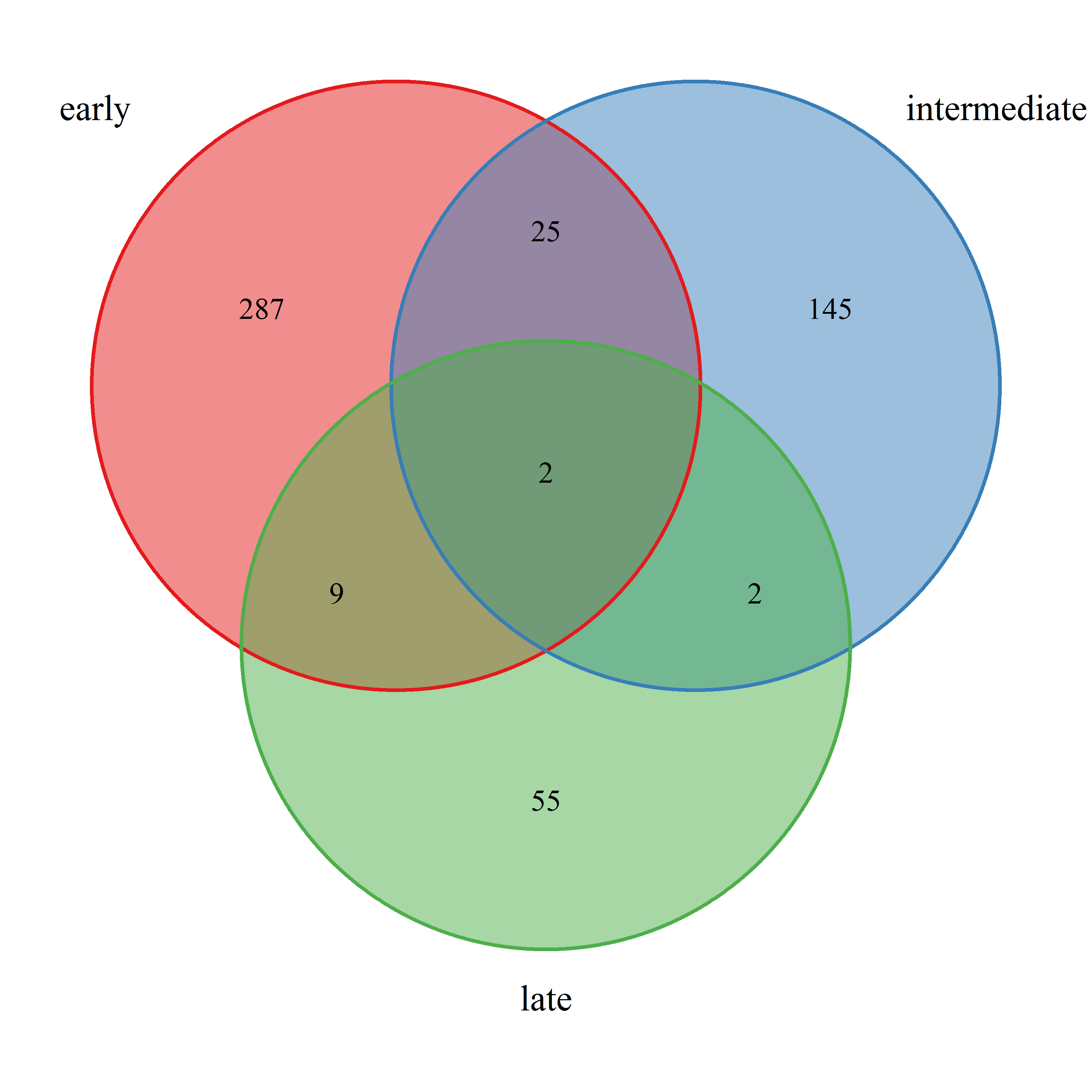


**Overlap of selected GO terms in group 2 (immunological tissues).** The three sets show the number of selected GO terms by the different variants of meta-analysis: GSEA on the merged data set (early merging), on the study-specific differential expression results (intermediate merging) and on the differential expression results after p-value combination (late merging). A p-value cutoff of 0.01 was used.
